# Coronavirus Non-Structural Protein 1 Is a Major Pathogenicity Factor: Implications for the Rational Design of Coronavirus Vaccines

**DOI:** 10.1371/journal.ppat.0030109

**Published:** 2007-08-10

**Authors:** Roland Züst, Luisa Cervantes-Barragán, Thomas Kuri, Gjon Blakqori, Friedemann Weber, Burkhard Ludewig, Volker Thiel

**Affiliations:** 1 Research Department, Kantonal Hospital St. Gallen, St. Gallen, Switzerland; 2 Unidad de Investigación Médica en Inmunoquímica, Hospital de Especialidades, Centro Médico Nacional Siglo XXI, Instituto Mexicano del Seguro Social, México City, México; 3 Department of Virology, University of Freiburg, Freiburg, Germany; National Institutes of Health, United States of America

## Abstract

Attenuated viral vaccines can be generated by targeting essential pathogenicity factors. We report here the rational design of an attenuated recombinant coronavirus vaccine based on a deletion in the coding sequence of the non-structural protein 1 (nsp1). In cell culture, nsp1 of mouse hepatitis virus (MHV), like its SARS-coronavirus homolog, strongly reduced cellular gene expression. The effect of nsp1 on MHV replication in vitro and in vivo was analyzed using a recombinant MHV encoding a deletion in the nsp1-coding sequence. The recombinant MHV nsp1 mutant grew normally in tissue culture, but was severely attenuated in vivo. Replication and spread of the nsp1 mutant virus was restored almost to wild-type levels in type I interferon (IFN) receptor-deficient mice, indicating that nsp1 interferes efficiently with the type I IFN system. Importantly, replication of nsp1 mutant virus in professional antigen-presenting cells such as conventional dendritic cells and macrophages, and induction of type I IFN in plasmacytoid dendritic cells, was not impaired. Furthermore, even low doses of nsp1 mutant MHV elicited potent cytotoxic T cell responses and protected mice against homologous and heterologous virus challenge. Taken together, the presented attenuation strategy provides a paradigm for the development of highly efficient coronavirus vaccines.

## Author Summary

Prevention of viral diseases by vaccination aims for controlled induction of protective immune responses against viral pathogens. Live viral vaccines consist of attenuated, replication-competent viruses that are believed to be superior in the induction of broad immune responses, including cell-mediated immunity. The recent proceedings in the area of virus reverse genetics allows for the rational design of recombinant vaccines by targeting, i.e., inactivating, viral pathogenicity factors. For coronaviruses, a major pathogenicity factor has now been identified. The effect of coronavirus non-structural protein 1 on pathogenicity has been analyzed in a murine model of coronavirus infection. By deleting a part of this protein, a recombinant virus has been generated that is greatly attenuated in vivo, while retaining immunogenicity. In particular, the mutant virus retained the ability to replicate in professional antigen-presenting cells and fulfilled an important requirement of a promising vaccine candidate: the induction of a protective long-lasting, antigen-specific cellular immune response. This study has implications for the rational design of live attenuated coronavirus vaccines aimed at preventing coronavirus-induced diseases of veterinary and medical importance, including the potentially lethal severe acute respiratory syndrome.

## Introduction

Coronaviruses are vertebrate pathogens mainly associated with respiratory and enteric diseases [1]. They can cause severe diseases in livestock animals and lead thereby to high economic losses. In humans, coronavirus infections manifest usually as mild respiratory tract disease (common cold) that may cause more severe symptoms in elderly or immune-compromised individuals [2,3]. In 2002–2003, the appearance of severe acute respiratory syndrome (SARS), caused by a formerly unknown coronavirus (SARS-CoV), exemplified the potential of coronaviruses to seriously affect human health [4–7]. The frequent detection of SARS-like coronaviruses in horseshoe bats (Rhinolophus sp.) and the broad range of mammalian hosts that are susceptible to SARS-CoV infection may facilitate a potential reintroduction into the human population [8]. Therefore, the development of efficacious coronavirus vaccines is of high medical and veterinary importance.

Effective vaccines controlling virus spread and disease are available for a number of infections, such as smallpox, poliomyelitis, measles, mumps, rubella, influenza, hepatitis A, and hepatitis B [9,10]. Some of these vaccines consist of virus subunits or inactivated virus preparations that mainly induce the production of pathogen-specific antibodies. In contrast, live attenuated vaccines consist of replication-competent viruses that induce broad cellular and humoral immune responses without causing disease [10]. The most prominent live attenuated vaccines are vaccinia virus [11], poliovirus [12], and yellow fever virus (YF-17D) [13]. Despite their documented efficacy, it is still not fully understood why and how successful vaccines work [10,14]. However, recent concepts in immunology provide a link between innate and adaptive immune responses and suggest that the quality, quantity, and longevity of adaptive immune responses is determined very early after infection or vaccination [14]. Of major importance are professional antigen-presenting cells (pAPCs) such as dendritic cells (DCs) and macrophages, which play a major role in (i) sensing pathogen-associated molecular patterns, (ii) inducing innate immune responses, and (iii) shaping the upcoming adaptive immune response. Efficient live attenuated vaccines should therefore not only lack significant pathogenicity, but should also deliver antigens to pAPCs and activate the innate immune system.

Notably, the majority of currently available attenuated vaccines have been derived empirically. Given the recent proceedings in the areas of virus reverse genetics and virus–host interactions, the time should be ripe for more rational approaches in vaccine development. An attractive strategy is to target virally encoded pathogenicity factors, such as interferon (IFN) antagonists [15], to attenuate virulence while retaining immunogenicity. This concept has been proposed for the generation of live attenuated influenza virus vaccines encoding altered NS1 proteins [16,17].

Our rudimentary knowledge on coronavirus-encoded pathogenicity factors is reflected by the fact that only a few putative coronaviral pathogenicity factors have been identified and that functional analyses are still limited to the description of in vitro effects [18–20]. For a number of reasons, the non-structural protein 1 (nsp1) is of particular interest in this context. First, coronaviruses are positive-stranded RNA viruses, and the replicase-encoded nsps are expressed from the viral genomic RNA immediately after virus entry by translation of two large polyproteins. nsp1 is encoded at the 5′ end of the replicase gene and is therefore the first mature viral protein expressed in the host cell cytoplasm [21]. Second, a recent in vitro study suggests that SARS-CoV nsp1 may be associated with host cell mRNA degradation and may counteract innate immune responses [18]. Finally, nsp1 is encoded by all mammalian coronaviruses known to date (coronavirus groups 1, 2a, and 2b) [22], and recent structural data on SARS-CoV (group 2b) nsp1 suggest functional similarities to mouse hepatitis virus (MHV; group 2a) nsp1 [23].

Using a reverse genetics approach, we show here that nsp1 is a major pathogenicity factor. Recombinant MHV mutants encoding a deletion in nsp1 replicated as efficiently as wild-type virus in cell culture, but displayed an unprecedented degree of attenuation in mice. Interference with the type I IFN system appears to be the dominant mode of action of murine coronavirus nsp1. Vaccination with the nsp1 mutant virus elicited efficient memory cytotoxic T cell responses and protected against homologous and heterologous virus infections. Our study will pave the way for the generation of novel coronavirus vaccines based on modified coronavirus replicase genes.

## Results

### Transient nsp1 Expression Affects Cellular Gene Expression

We assessed several replicase-encoded nsps of MHV (strain A59), SARS-CoV, and human coronavirus 229E (HCoV-229E) for their ability to interfere with host cell gene expression. Using transient gene expression studies, we found that MHV-A59, SARS-CoV, and HCoV-229E nsp1 significantly reduced luciferase reporter gene expression under the control of IFN-β, IFN-stimulated response element (ISRE), and SV40 promoters (Figure 1). This is consistent with a recent report suggesting that SARS-CoV nsp1 induces general host cell mRNA degradation [18]. Nevertheless, it should be noted that the nsp1-mediated reduction in reporter protein expression appeared more robust for ISRE and SV40 than for IFN-β promoter-driven expression. Our data also support the hypothesis that MHV encodes a SARS-CoV nsp1 homolog that displays the same function [23]. Although comparative sequence analyses suggest that nsp1 of group 2a/2b coronaviruses (e.g., MHV and SARS-CoV, respectively) and the nsp1 of group 1 coronaviruses (e.g., HCoV-229E) may belong to different protein families [22,23], we also observed reduced reporter gene expression in HCoV-229E nsp1-transfected cells (Figure 1). Whether functional similarities may exist between nsp1 molecules encoded by coronaviruses of different phylogenetic lineages remains to be established in future studies. Importantly, our data also revealed that reporter gene expression from all tested promoters was not affected when C-terminally truncated MHV nsp1 molecules were tested (Figure 1).

**Figure 1:**
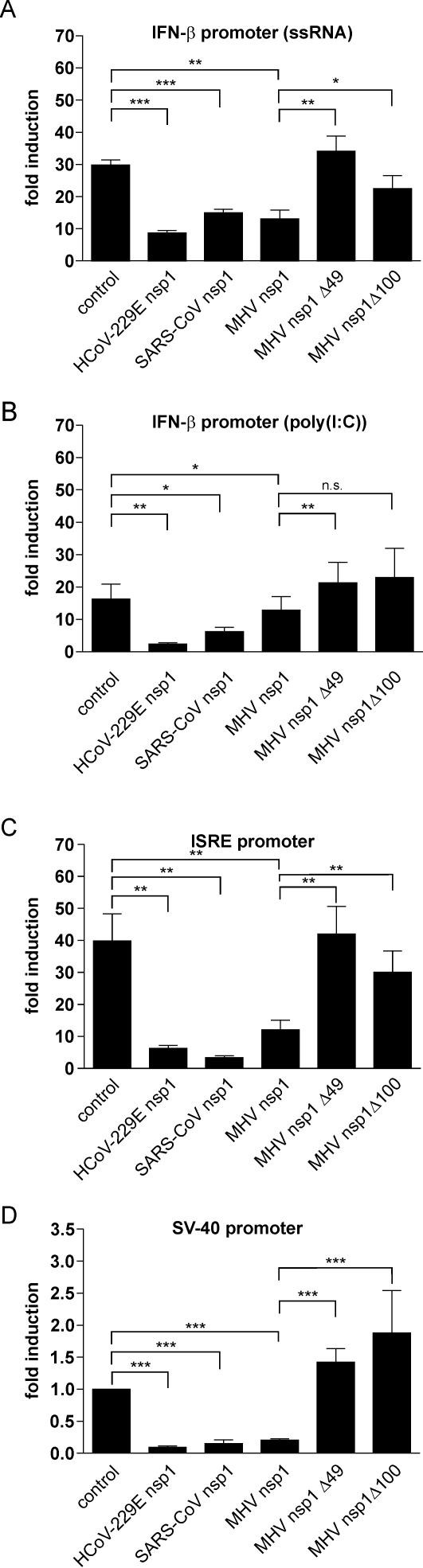
Coronavirus nsp1 Reduces Cellular Gene Expression (A–C) 293 cells were transfected with p125-Luc reporter plasmid ([A, B]; IFN-β promoter), or p(9–27)4tkD(−39)lucter reporter plasmid ([C]; ISRE promoter) and cotransfected with pRL-SV40, and an expression plasmid encoding a full-length coronavirus nsp1 of HCoV-229E, SARS-CoV, or MHV, or a truncated MHV nsp1 variant (49-nt or 100-nt 3′ end truncations, respectively). At 8 h post transfection, cells were treated with viral single-stranded RNA containing 5′ triphosphates (A) or poly(I:C) (B), or IFN-α (C), and 16 h later luciferase activity was measured. Firefly luciferase activity was normalized to renilla luciferase activity and indicated as fold induction compared to that of control plasmid-transfected cells (control). (D) 293 cells were transfected with pRL-SV40 and the indicated expression plasmids. Then, 24 h post transfection, luciferase activity was measured. Results represent the mean ± SD of at least four independent experiments. Statistical analysis was performed using paired Student's *t*-test (***, *p* < 0.001; **, *p* < 0.01; *, *p* < 0.05; n.s. (not significant), *p* > 0.05).

### Generation of the MHV nsp1 Deletion Mutant

To assess the role of nsp1 in the context of virus replication, we constructed a recombinant MHV encoding a truncated nsp1 protein using our reverse genetic system [24]. Based on the results shown in Figure 1, we decided to delete MHV nucleotides (nts) 829–927 (99 nts). In the resulting mutant virus, MHV-nsp1Δ99, the replicase gene start codon, the translational reading frame, and the residues required for proteolytic release of nsp1 from the replicase polyprotein were maintained (Figure 2A). As reported for a set of similar MHV mutants by Brockway et al. [25], viral growth and peak titers of MHV-nsp1Δ99 in murine 17Clone1 cells were indistinguishable from that of wild-type virus (Figure 2B). To assess the stability of the recombinant MHV-nsp1Δ99, we analyzed the nsp1-coding region by RT-PCR sequencing after seven passages in tissue culture and no nucleotide changes were detected (unpublished data).

**Figure 2:**
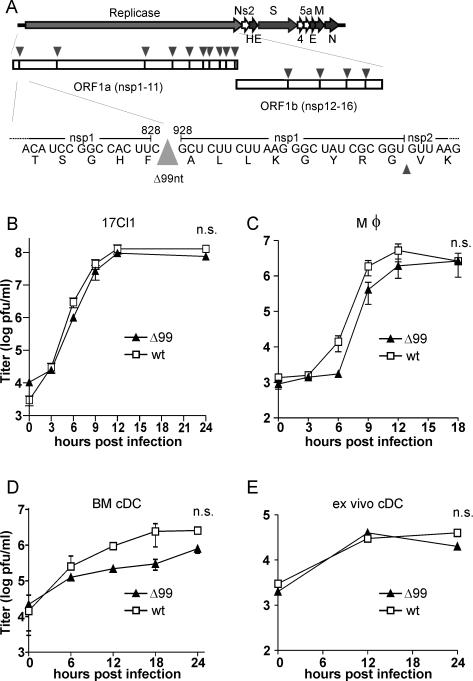
Construction and In Vitro Analysis of MHV-nsp1Δ99 (A) Schematic representation of the MHV-nsp1Δ99 genome organization. The replicase gene, comprised of open reading frames (ORFs) 1a and 1b, is depicted together with viral proteinase cleavage sites (arrowheads) that separate nsps 1–16. The 99-nt deletion within the nsp1-coding region of MHV-nsp1Δ99 is illustrated on the nucleotide and amino acid level. The arrowhead (far right) indicates the nsp1/nsp2 cleavage site. (B–E) Growth kinetics of MHV-nsp1Δ99– or MHV-A59–infected (MOI = 1) murine 17Clone1 cells (B), inflammatory macrophages (C), bone marrow–derived cDCs (D), and ex vivo cDCs (E). Experiments (C–E) were performed with cells obtained from C57BL/6 mice. Results represent the mean ±SD of two independent experiments. Statistical analysis was performed using Student's *t*-test (n.s., *p* > 0.05).

Infection of conventional DCs (cDCs) is an early and crucial event for the generation of protective antiviral immunity [26]. MHV productively infects cDCs and activates plasmacytoid DCs (pDCs) to generate a first wave of protective type I IFN [27]. To assess whether the mutant MHV-nsp1Δ99 has retained the ability to infect pAPCs, peritoneal macrophages (Figure 2C), bone marrow–derived CD11c^+^ cDCs (Figure 2D), and splenic, FACS-sorted CD11c^+^ cDCs (Figure 2E) were exposed to MHV-nsp1Δ99 and wild-type control virus. Similar to replication kinetics in cell lines (Figure 2B), MHV-nsp1Δ99 showed no significant growth defect in primary pAPCs (Figure 2C–2E), indicating that the deletion of nsp1 did not alter the pronounced tropism of MHV for cDCs and macrophages.

### Deletion in nsp1 Confers Strong Attenuation In Vivo

MHV-A59 is a hepatotropic and neurotropic virus that can cause acute hepatitis and encephalitis. Following intraperitoneal infection, virus replication is first detectable in spleen and liver, followed by virus spread to other organs, including the central nervous system. Hepatitis is the first clinical sign of disease, accompanied by elevated liver enzyme values in serum. Associated with the appearance of cytotoxic T cell responses approximately at day 5 post infection (p.i.), virus titers usually decline and are no longer detectable after day 7 p.i. Infections with a high dose (≥ 5 × 10^6^ pfu, intraperitoneal) may, however, occasionally result in fatality. To evaluate the importance of nsp1 for virus replication and viral pathogenicity in vivo, C57BL/6 mice were infected intraperitoneally with different doses of wild-type MHV or MHV-nsp1Δ99. Both viruses replicated in the spleen, whereby MHV-nsp1Δ99 titers were consistently lower than wild-type virus titers (Figure 3A). Furthermore, MHV-nsp1Δ99 was rapidly cleared and not detectable after day 2 p.i. (Figure 3A). Wild-type, but not mutant virus, was detectable in the liver at low and intermediate dose (50 pfu and 5,000 pfu, respectively) (Figure 3B). When high virus doses (5 × 10^6^ pfu) were applied, MHV-nsp1Δ99 eventually reached the liver at day 2 p.i., but was not detectable at later time points (Figure 3B). MHV-nsp1Δ99 was not detectable in other non-hematopoietic organs, such as lung and central nervous system (unpublished data). Mice infected with wild-type virus showed acute liver disease with elevated liver enzyme values in serum. Furthermore, after high dose infection with wild-type virus (5 × 10^6^ pfu), a significant weight loss that peaked at approximately 10%–15% at day 4 was observed (Figure 3E). In contrast, mice infected with the nsp1 mutant virus remained healthy after low, intermediate, or high dose infections. Even at the highest dose applied (5 × 10^6^ pfu), MHV-nsp1Δ99–infected mice did not lose weight (Figure 3E), and no elevated liver enzyme values were detected in the serum (Figure 3C). This observation correlated well with the absence of hepatocyte necrosis and parenchymal inflammation following MHV-nsp1Δ99 infection (Figure 3D). To further assess the attenuation of the MHV nsp1 mutant, mice were infected intracranially with 200 pfu and 20,000 pfu of MHV-nsp1Δ99 or MHV-A59. All mice infected with 200 pfu survived for at least 30 d (unpublished data). Mice infected with 20,000 pfu of MHV-A59 succumbed to the infection, whereas mice infected with 20,000 pfu of MHV-nsp1Δ99 survived and showed no signs of clinical disease (Figure 3F). Collectively, these data demonstrate that MHV-nsp1Δ99 is strongly attenuated in vivo, but has retained the ability to replicate in secondary lymphoid organs, such as the spleen.

**Figure 3:**
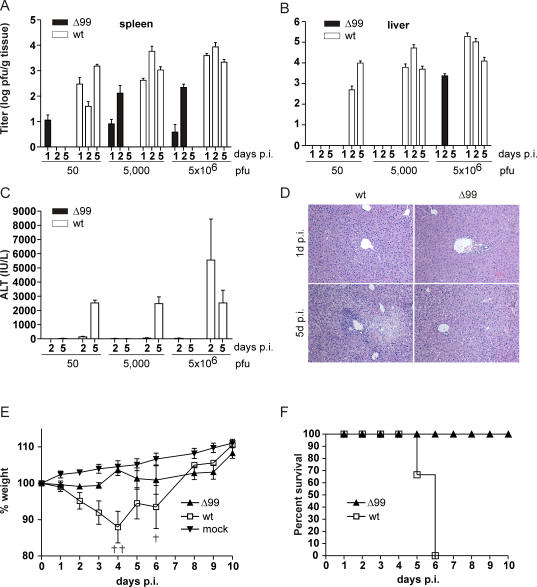
MHV-nsp1Δ99 Is Highly Attenuated In Vivo C57BL/6 mice were infected intraperitoneally (A–E) or intracranially (F) with the indicated dose of MHV-nsp1Δ99 or MHV-A59. Viral titers in spleens (A) and livers (B) were determined at the indicated time points p.i. (C) Liver enzyme ALT values were measured at the indicated time points p.i. (D) Hematoxylin and eosin–stained liver sections of C57BL/6 mice infected intraperitoneally with 5,000 pfu of MHV-nsp1Δ99 or MHV-A59. The time points of analysis p.i. are indicated. (E) Groups of four mice were either untreated (mock) or infected (intraperitoneally) with 5 × 10^6^ pfu of MHV-nsp1Δ99 or MHV-A59. The weight was monitored daily. † denotes death of mice. (F) Survival of mice (three per group) infected with 20,000 pfu (intracranially) of MHV-nsp1Δ99 or MHV-A59. Mice with severe weight loss (>25%) were defined as moribund and sacrificed. Results (A–C) and (E) represent the mean ±SD of at least three individual mice per time point.

### Effect of nsp1 on Innate Immune Responses

We have previously shown that pDCs are the major source of IFN-α in the early stages of MHV infection and that type I IFN responses in CD11c^+^ cDCs are only weakly triggered by MHV [27]. To test whether nsp1 has an influence on the induction of IFN-α, we infected both cDCs and pDCs with MHV-nsp1Δ99 or wild-type MHV. Both viruses elicited rapid and high IFN-α production in Flt3-L–differentiated bone marrow–derived pDCs (Figure 4A) and FACS-sorted primary pDCs (Figure 4B). Furthermore, both wild-type and mutant MHV elicited only a late and weak IFN-α production in cDCs (Figure 4A and 4B). These results suggest that nsp1 does not affect the induction of type I IFN. To assess a potential impact of nsp1 on type I IFN signaling and antiviral effector mechanisms in target cells that efficiently support MHV replication, cDCs and macrophages were pretreated with different dosages of IFN-α and infected with MHV-nsp1Δ99 or wild-type MHV. In cDCs, IFN-α treatment had a comparable effect on the replication of both MHV-nsp1Δ99 and the wild-type control virus (Figure 4C). However, replication of MHV-nsp1Δ99 was, in a dose-dependent manner, more vulnerable to IFN-α treatment in macrophages (Figure 4D), suggesting that nsp1 might counteract IFN signaling and/or the antiviral activities of IFN-induced effector proteins.

**Figure 4:**
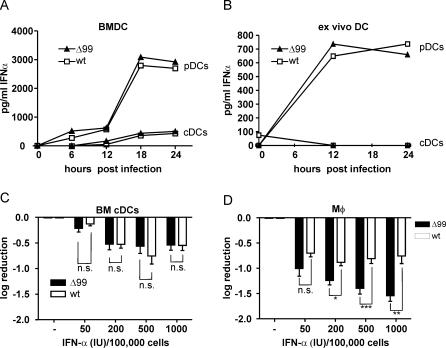
Effect of nsp1 on IFN-α Production and Signaling C57BL/6 bone marrow–derived (A) or primary (B) splenic cDCs or pDCs were infected with MHV-nsp1Δ99 or MHV-A59 at an MOI of 1. IFN-α secreted into cell culture supernatants was determined by ELISA at the indicated time points. Bone marrow–derived cDCs (C) or inflammatory macrophages (D) from 129Sv mice were treated with 50, 200, 500, or 1,000 U IFN-α/100,000 cells or left untreated. Four hours later, cells were infected with MHV-nsp1Δ99 or MHV-A59 (MOI = 1). Twelve hours p.i., virus titers in culture supernatants were determined by plaque assay. Representative experiments out of two (A, B) or the mean ± SD of two independent experiments (C, D) are shown. Statistical analysis was performed using Student's *t*-test (***, *p* < 0.001; **, *p* < 0.01; *, *p* < 0.05; n.s., *p* > 0.05).

Indeed, in vivo experiments in type I IFN receptor-deficient (IFNAR^−/−^) mice [28] strongly support this interpretation. Infection of IFNAR^−/−^ mice with wild-type MHV led to high titers in all tested organs (Figure 5A–5D), indicating that signals transmitted via the IFNAR are crucial for preventing uncontrolled spread of the virus [27]. Surprisingly, the severe attenuation of MHV-nsp1Δ99 in wild-type 129Sv mice was not present in IFNAR^−/−^ mice (Figure 5A–5D). Replication of MHV-nsp1Δ99 in IFNAR^−/−^ mice was largely restored and virus titers reached about 10^4^–10^5^ pfu/g tissue in several organs after only 36 h (figures 5A–5D). These data strongly suggest that nsp1 has a pivotal role in counteracting type I IFN host responses and provide an explanation for the rapid clearance of MHV-nsp1Δ99 in wild-type mice. Interestingly, liver damage, measured as alanine 2-oxoglutarate-aminotransferase (ALT) levels in serum, was not yet detectable in MHV-nsp1Δ99–infected IFNAR^−/−^ mice at 36 h p.i. (Figure 5E). At 72 h p.i., MHV-nsp1Δ99 reached titers and ALT levels in IFNAR^−/−^ mice comparable to those observed in MHV-A59–infected IFNAR^−/−^ mice at 36 h p.i., demonstrating that MHV-nsp1Δ99 replication in IFNAR^−/−^ is, although with slower kinetics, restored.

**Figure 5:**
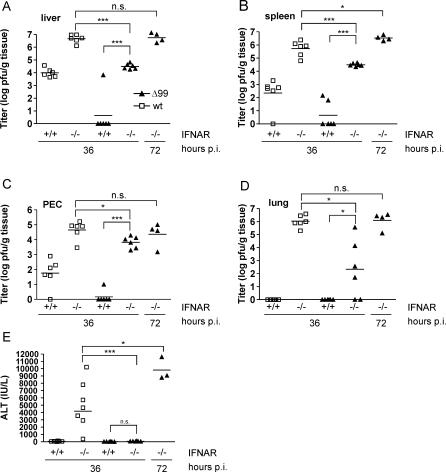
nsp1-Dependent Attenuation Is Reversed in IFNAR^−/−^ Mice IFNAR^−/−^ or wild-type 129Sv mice were infected intraperitoneally with 500 pfu of MHV-nsp1Δ99 or MHV-A59. At the indicated time points p.i., viral titers in livers (A), spleens (B), peritoneal exudates cells (PEC) (C), and lungs (D) were determined. (E) ALT values in serum were measured at the indicated time points p.i. Horizontal lines represent means with values from individual mice shown as open squares (MHV-A59) or filled triangles (MHV-nsp1Δ99) from two experiments with a total of three to six mice. Statistical analysis was performed using Student's *t*-test (_***_, *p* < 0.001; _*_, *p* < 0.05; n.s., *p* > 0.05).

### Immunization with the MHV nsp1 Deletion Mutant Protects against Homologous and Heterologous Virus Challenge

The phenotypic analysis of MHV-nsp1Δ99 revealed a number of features that are advantageous for live attenuated vaccines. MHV-nsp1Δ99 grows to high titers in cell culture, infects pAPCs, replicates almost exclusively in secondary lymphoid organs, and is strongly attenuated in vivo. To assess the potential of MHV-nsp1Δ99 as an attenuated live vaccine, we replaced accessory gene 4 of MHV-nsp1Δ99 and wild-type MHV-A59 by a gene encoding a fusion protein of the immunodominant cytotoxic T lymphocyte (CTL) epitope (KAVYNFATC) of the lymphocytic choriomeningitis virus (LCMV) and the enhanced green fluorescent protein (GP33-GFP) [29]. The resulting recombinant viruses, MHV-nsp1Δ99-GP33-GFP and MHV-GP33-GFP, were used to infect C57BL/6 mice with different doses (50 and 5,000 pfu, intraperitoneal), and CD8^+^ T cell responses were assessed using flow cytometry–based detection of intracellular IFN-γ following antigen-specific short-term in vitro restimulation. As shown in Figure 6A and 6B, infection with as few as 50 pfu of MHV-nsp1Δ99-GP33-GFP elicited strong CD8^+^ T cell responses against both the H2-D^b^–restricted GP33 and the H2-K^b^–restricted MHV S598 epitope.

**Figure 6:**
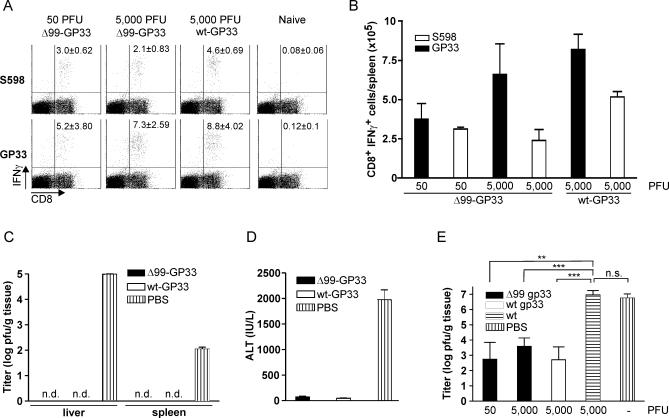
MHV-nsp1Δ99-GP33-GFP and MHV-GP33-GFP Elicit Strong CTL Responses and Protect Mice from Homologous and Heterologous Viral Infections (A, B) Groups of three C57BL/6 mice were immunized with the indicated doses of MHV-nsp1Δ99-GP33-GFP or MHV-GP33-GFP. IFN-γ–secreting CD8^+^ splenocytes were determined 8 d post immunization following short-term in vitro restimulation with GP33 or S598 peptide. Values in (A) represent the percentage ±SD of IFN-γ–secreting CD8^+^ T cells restimulated with GP33 or S598; values in (B) represent the absolute numbers of CD8^+^ IFN-γ^+^ cells ±SD (*n* = 6 for GP33, *n* = 3 for S598). Pooled data from two separate experiments are shown. (C, D) Groups of three mice were immunized with 5,000 pfu of the indicated virus or PBS-treated and 14 d later challenged with 5,000 pfu of wild-type MHV-A59. At 5 d post challenge, viral titers in liver and spleen were determined (C) and ALT values (D) were measured. Data in graphs (C) and (D) represent means ±SD from one representative experiment. n.d., not detected. (E) Protection against heterologous viral challenge. Groups of four C57BL/6 mice have been immunized with the indicated viral doses or PBS-treated and 28 d later challenged with 200 pfu of LCMV-WE. At 4 d post challenge, viral titers in spleens were determined. Data in graph represent means ±SD from two pooled experiments with a total of eight mice per group. Statistical analysis was performed using Student's *t*-test (***, *p* < 0.001; **, *p* < 0.01; n.s., *p* > 0.05).

To assess the level of protection against homologous MHV-A59 challenge, groups of C57BL/6 mice were immunized (5,000 pfu) with MHV-nsp1Δ99-GP33-GFP, MHV-GP33-GFP, or treated with PBS. Sixteen days p.i., mice were challenged with wild-type MHV (5,000 pfu) and viral titers were determined 5 d post challenge infection. Viral titers were below the limit of detection in MHV-nsp1Δ99-GP33-GFP– and MHV-GP33-GFP–immunized mice (Figure 6C). Together with the absence of elevated liver enzyme values in immunized mice (Figure 6D), these data indicate that vaccination with the attenuated MHV nsp1 mutant provides complete protection against homologous virus challenge.

The reverse genetic system facilitates incorporation of antigens derived from other infectious organisms. In order to determine whether the attenuated nsp1 mutant virus could confer protection against heterologous virus infection, MHV-nsp1Δ99-GP33-GFP–immunized C57BL/6 mice were challenged after 4 wk with LCMV (200 pfu, intravenous). LCMV titers in spleens were significantly reduced both in mice vaccinated with MHV-GP33-GFP and the attenuated MHV-nsp1Δ99-GP33-GFP virus (Figure 6E). Remarkably, only 50 pfu of nsp1 mutant virus expressing the GP33 epitope were sufficient to achieve a reduction of LCMV titers by more than 4 orders of magnitude, indicating that nsp1 mutant viruses are well-suited to serve as attenuated recombinant virus vectors against heterologous viral infections.

## Discussion

The rational design of live attenuated viral vaccines is greatly facilitated by the identification and targeting of pathogenicity factors. This study demonstrates an unprecedented level of attenuation of a murine coronavirus through a 99-nt deletion in nsp1. The nsp1 mutant virus was rapidly cleared in mice and did not induce clinical signs of disease in immunocompetent mice. These findings in the murine coronavirus model demonstrate that nsp1 is a major pathogenicity factor. In a stepwise approach, we made use of these observations to provide a blueprint for the construction and evaluation of live attenuated coronavirus vaccines encoding a truncated nsp1.

The presented results indicate that nsp1 plays a crucial role in the MHV life cycle by interfering with host innate immune responses. In accordance with the recent report by Kamitani et al. [18], we observed reduced reporter gene expression in transient nsp1 expression studies. The suggestion that SARS-CoV nsp1 may play a role in SARS-CoV pathogenesis by promoting host cell mRNA degradation [18] has now received support through the analysis of a coronavirus nsp1 mutant in a murine model. The MHV nsp1 mutant phenotype led us to conclude that nsp1 mainly affects IFN signaling pathways or other downstream events. The influence on IFN-α induction appears to be limited. These conclusions are based on several observations. First, the analysis of IFN-α production by pDCs and cDCs revealed no significant differences between wild-type and nsp1 mutant virus infections. Second, treatment of macrophages with IFN-α revealed a more efficient reduction of MHV-nsp1Δ99 replication compared with that of wild-type MHV. Finally, and most strikingly, IFNAR^−/−^ mice were highly permissive for the mutant virus, and organ titers almost reached those of wild-type MHV-infected IFNAR^−/−^ mice. Nevertheless, it should be noted that the nsp1 mutant virus replication was still slightly delayed in IFNAR^−/−^ mice. Therefore, further studies are required to define molecular target(s) and the precise function(s) of coronavirus nsp1. Likewise, further studies are required to assess the impact of other coronaviral gene products on coronavirus pathogenicity. Recent reports indicate that coronaviruses most likely express a number of proteins, such as MHV and SARS-CoV nucleocapsid proteins, and SARS-CoV ORF3b and ORF6 proteins, that may interact with innate immune responses [19,20]. Also, the coronavirus replicase gene may harbor additional functions that play a role in virus–host interactions. It has been shown that the MHV and SARS-CoV nsp2 proteins [30], and the highly conserved ADP-ribose-1′′-monophosphatase activity [31] encoded in nsp3, are both dispensable for virus replication in tissue culture, and that a single point mutation in the MHV nsp14 confers a strong attenuation of MHV in mice [32]. Clearly, the murine model, with MHV as a natural mouse pathogen, will be highly advantageous in the examination of this issue, because it allows the use of well-characterized inbred and transgenic mice in combination with well-established immunological techniques required to assess the full range of coronavirus–host interactions.

The most remarkable finding of this study is the level of attenuation of the nsp1 mutant virus and its restricted replication in secondary lymphoid organs. It may well be that other coronaviral nsp1 molecules exert similar functions as the MHV nsp1. The coronavirus nsp1 has been suggested as a group-specific marker to differentiate group 1 coronaviruses from group 2a/2b coronaviruses [22]. Our transient nsp1 expression data indeed support the notion that SARS-CoV and MHV may encode evolutionarily conserved nsp1 homologs [22,23]. Nevertheless, further in vivo studies are required to determine whether the group 2b SARS-CoV nsp1 is indeed a functional equivalent to the structurally highly conserved group 2a nsp1 molecules encoded by MHV, bovine coronavirus, porcine hemagglutinating encephalomyocarditis virus, HCoV-OC43, and HCoV-HKU1. Likewise, it will be important to clarify in vivo, whether, despite the apparent lack of any sequence homology [22,23], the nsp1 of group 1 coronaviruses (e.g., HCoV-229E) may represent a functional correlate to the nsp1 of group 2a/2b coronaviruses. Recent progress in the establishment of suitable mouse models for SARS-CoV [33–35] and HCoV-229E [36] will enable researchers to address these questions in future studies.

The chosen attenuation strategy has resulted in the generation of a recombinant virus that fulfills important criteria of a live virus vaccine candidate: (i) growth to high titers in cell culture, which facilitates vaccine production, and (ii) generation of immunological memory that mediates efficient protection against viral challenge. One important aspect of protection against viral infections is the induction of specific cytotoxic T cells by pAPCs in secondary lymphoid organs [14]. A number of coronaviruses, such as MHV, HCoV-229E, feline infectious peritonitis virus, and SARS-CoV, have been shown to infect pAPCs and to replicate in the secondary lymphoid organs [27,37–42]. Because of their pronounced tropism for pAPCs and the induction of strong CTL responses, we propose that coronaviruses represent promising vectors for the expression of heterologous antigens. The identification of nsp1 as a major pathogenicity factor will significantly increase the safety of coronavirus-based vectors [43]. For example, the deletion of accessory genes (i.e., not replicase or structural genes) has been described for some coronaviruses to confer attenuation in the natural host [44–46], and the deletion of the structural envelope protein E has resulted in the development of replication-competent, but propagation-deficient, coronavirus vectors [47,48]. Now, with an accompanying deletion in the nsp1-coding sequence, such vectors would be considered “recombination proof”, because the deletions are located at opposite genomic regions (i.e., within the replicase gene at the 5′ end and within the structural gene region at the 3′ end of the coronavirus genome), which make the reconstruction of virulent viruses by recombination unlikely. We therefore suggest that accessory gene, E gene, and partial nsp1 gene deletions will result in particular safe vectors with the potential to express multiple heterologous antigens [40,49].

Taken together, our results describe a novel type of coronavirus vaccines based on impaired function of a replicase gene product. We expect that our approach is applicable to most, if not all, mammalian coronaviruses and that it will enable the development of long-desired live attenuated vaccines for important coronavirus-induced diseases in humans and animals.

## Materials and Methods

### Mice and cells.

C57BL/6 mice were obtained from Charles River Laboratories (http://www.criver.com/). 129Sv and type I IFN receptor-deficient mice (IFNAR^−/−^) [28] were obtained from the Institut für Labortierkunde (University of Zürich) and bred in our facilities. All mice were maintained in individually ventilated cages and were used between 6 and 9 wk of age. All animal experiments were performed in accordance with the Swiss Federal legislation on animal protection.

MC57, BHK-21, L929, 293, and CV-1 cells were purchased from the European Collection of Cell Cultures (http://www.ecacc.org.uk/). D980R cells were a kind gift from G. L. Smith, Imperial College, London, United Kingdom. 17Clone1 cells were a kind gift from S. G. Sawicki, Medical University of Ohio, Toledo, Ohio, United States. BHK-MHV-N cells, expressing the MHV-A59 nucleocapsid protein under the control of the TET/ON system (Clontech, http://www.clontech.com/), have been described previously [24]. All cells were maintained in minimal essential medium supplemented with fetal bovine serum (5%–10%) and antibiotics.

### Isolation of dendritic cells and macrophages, flow cytometry, and antibodies.

Murine cDCs and pDCs were obtained from spleens of C57BL/6, 129Sv, or IFNAR^−/−^ mice following digestion with collagenase type II (Gibco-BRL, http://www.invitrogen.com/) for 20 min at 37 °C. Cells were resuspended in PBS supplemented with 2% FCS and 2 mM EDTA and overlaid on 20% Optiprep density gradient medium (Sigma-Aldrich, http://www.sigmaaldrich.com/). After centrifugation at 700*g* for 15 min, low density cells were depleted of CD3- and CD19-positive cells using DYNAL magnetic beads according to the instructions of the manufacturer (Invitrogen, http://www.invitrogen.com/). The DC-enriched preparations were stained with α-PDCA-1, α-CD11b, and α-CD11c, and the distinct pDC and cDC populations were sorted using a FACS ARIA (BD Biosciences, http://www.bdbiosciences.com/) sorter. Purity of both cell preparations was always >98%.

Murine bone marrow–derived cDCs or pDCs were generated by 6 to 7 d of culture with either granulocyte-monocyte colony stimulating factor (GM-CSF)-containing supernatant from the cell line X63-GM-CSF (kindly provided by Antonius Rolink, University of Basel, Basel, Switzerland) or Flt3-L (R&D Systems, http://www.rndsystems.com/) at 20 ng/ml, respectively. Bone marrow–derived cDCs were further purified using Optiprep density gradient centrifugation. Bone marrow–derived pDCs were purified using the mouse pDC isolation kit (Miltenyi Biotec, http://www.miltenyibiotec.com/) adapted for the isolation of in vitro–derived pDCs by adding CD11b-biotin to the negative selection cocktail. Antibodies used in this study were purchased from BioLegend (http://www.biolegend.com/): CD11c-PE, B220-APC, CD11b-FITC; or from Miltenyi Biotec: mPDCA-1-FITC and CD11c-APC. Thioglycolate-elicited macrophages were collected from the peritoneal cavity of mice and cultured at 4 × 10^5^ cells per well in DMEM (with 10% FCS, L-glutamine, and penicillin/streptomycin) for 2 h at 37 °C. Non-adherent cells were removed by washing with cold PBS.

### Recombinant DNA and viruses.

LCMV-WE strain, originally obtained from F. Lehmann-Grube (Hamburg, Germany), was propagated on L929 cells. MHV A59 was generated from a molecularly cloned cDNA [24] based on the Albany strain of MHV A59. Coronaviruses and recombinant vaccinia viruses were propagated, titrated, and purified as described [24,50,51].

Mutant vaccinia viruses are based on the recombinant vaccina virus vMHV-inf-1 (containing the full-length MHV-A59 cDNA) and were generated using our reverse genetic system as described previously [24]. Briefly, the gene to be mutated was replaced by the *Escherichia coli* guanine-phosphoribosyltransferase (GPT) gene through vaccinia virus–mediated homologous recombination, and GPT-positive clones were selected by three rounds of plaque purification on CV-1 cells in the presence of xanthine, hypoxanthine, and mycophenolic acid (GPT-positive selection) [50]. In a second round, the GPT gene was replaced by the mutated gene, and GPT-negative clones, containing the mutated gene, were selected by three rounds of plaque purification on D980R cells in the presence of 6-thioguanine (GPT-negative seletion) [50]. To construct the recombinant vaccinia virus encoding the MHV-nsp1Δ99 cDNA, the 5′ end of the MHV-A59 cDNA in vMHV-inf-1 was replaced by GPT using the plasmid pRec4. This plasmid is based on pGPT-1 [50], and the GPT gene is flanked to its left by 500 bp of vaccinia DNA and to its right by an internal ribosomal entry sequence (IRES) followed by MHV-A59 nts 952-1315. The GPT-negative selection was carried out using the plasmid pRec15. This plasmid contains 250 bp of vaccinia DNA followed by the bacteriophage T7 RNA polymerase promoter, one G nucleotide, and MHV nts 1–828 linked to MHV nts 928-1315. To replace the MHV-A59 accessory gene 4 in vMHV-inf-1 and vMHV-nsp1Δ99 by a gene encoding a fusion protein of EGFP and the LCMV-derived CTL epitope KAVYNFATC (GP33-GFP) [29], the plasmid pRec8 was used for recombination with vaccinia viruses vMHV-inf-1 and vMHV-nsp1Δ99 . This plasmid contains the GPT gene flanked to its left by MHV nts 27500–27967 and to its right by MHV nts 28265–28700. GPT-negative selection was carried out using the plasmid pRec9. This plasmid contains MHV bp 27500–27967, the GP33-GFP gene, and MHV nts 28265–28700. Further cloning details, plasmid maps, and sequences are available from the authors upon request. Recombinant coronaviruses were rescued from cloned cDNA using purified vaccinia virus DNA as template for the in vitro transcription of recombinant MHV genomes as described [51].

The firefly luciferase (FF-Luc) plasmid for monitoring IFN-β promoter activation (p125-Luc) was kindly provided by Takashi Fujita, Tokyo Metropolitan Institute of Medical Science, Japan [52]. The FF-Luc reporter construct for monitoring ISRE activation (p(9–27)4tkΔ(−39)lucter) [53] was kindly provided by Stephen Goodbourn, St. George's Hospital Medical School, London, UK. The control plasmid pRL-SV40 (Promega, http://www.promega.com/) encodes the renilla luciferase (REN-Luc) gene under control of the constitutive SV40 promoter. The negative control expression plasmid contained the reading frame of the N-terminus of the human MxA protein. To construct the coronavirus nsp1 expression plasmids, the MHV nts 1–951 (pMHV-nsp1), MHV nts 1–902 (pMHV-Δ49), MHV nts 1–851 (pMHV-Δ100), HCoV-229E nts 293–625 (pHCoV-229E-nsp1), and SARS-CoV nts 265–804 (pSARS-CoV-nsp1) were amplified by standard PCR techniques and cloned downstream of a CMV promoter between the SmaI and XhoI sites of the eukaryotic high-level expression plasmid pI.18 (kindly provided by Jim Robertson, National Institute for Biological Standards and Control, Hertfordshire, UK).

### Transient transfections and reporter gene assays.

Subconfluent cell monolayers of 293 cells seeded in 12-well plates were transfected with 250 ng p125-Luc reporter plasmid, 50 ng pRL-SV40, and 1 μg of expression plasmid in 200 μl of OPTIMEM (Gibco-BRL) containing 3.9 μl of Fugene HD (Roche, http://www.roche.com). At 8 h post transfection, cells were induced with either 0.2 μg of viral ssRNA containing 5′ triphosphates [54] (p125-Luc), 2.5 μg of poly(I:C) (Sigma), or 500 U/ml IFN-α (p(9–27)4tkΔ(−39)lucter), or left uninduced. After an incubation period of 16 h, cells were harvested and lysed in 100 μl of Reporter Lysis Buffer (Promega). An aliquot of 10 μl lysate was used to measure luciferase activity as decribed by the manufacturer (Promega).

### Virus infections, determination of virus titers, and liver enzyme values.

Mice were injected intraperitoneally or intracranially with indicated pfu of MHV A59 or intravenously with the indicated pfu of LCMV and sacrificed at the indicated time points. Organs were stored at −70 °C until further analysis. Blood was incubated at RT to coagulate, centrifuged, and serum was used for ALT measurements using a Hitachi 747 autoanalyzer (http://www.hitachi.com/). Peritoneal exudates cells (PECs) were isolated from the peritoneal cavity by flushing with 4 ml of ice-cold PBS. MHV titers were determined by standard plaque assay using L929 cells. LCMV titers in the spleens were determined 4 d after intravenous challenge in an LCMV infectious focus assay as previously described [55].

### Histology, IFN-α ELISA, IFN-α treatment.

Organs were fixed in 4% formalin and embedded in paraffin. Sections were stained with hematoxylin and eosin. Images were acquired using a Leica DMRA microscope (Leica, http://www.leica.com/) with a 25×/0.65 NA objective (total magnification, ×162). Images were processed using Adobe Photoshop (Adobe Systems, http://www.adobe.com). Mouse IFN-α concentration in cell culture supernatants was measured by ELISA (PBL Biomedical Laboratories, http://www.interferonsource.com/) according to the manufacturer's instructions. IFN-α treatment of cells prior to MHV infection was performed using universal type I IFN (IFN-αA/D, Sigma).

### Intracellular cytokine staining.

Specific ex vivo production of IFN-γ was determined by intracellular cytokine staining. Organs were removed at the indicated time points following infection with recombinant MHV. For intracellular cytokine staining, single cell suspensions of 1 × 10^6^ splenocytes were incubated for 5 h at 37 °C in 96-well round-bottom plates in 200 μl of culture medium containing 25 U/ml IL-2 and 5 μg/ml Brefeldin A (Sigma). Cells were stimulated with phorbolmyristateacetate (PMA, 50 ng/ml) and ionomycin (500 ng/ml) (both purchased from Sigma) as positive control or left untreated as a negative control. For analysis of peptide-specific responses, cells were stimulated with 10^−6^ M GP33 peptide or 10^−4 ^ M MHV S598 peptide. The percentage of CD8^+^ T cells producing IFN-γ was determined using a FACSCalibur flow cytometer (BD Biosciences). Both S598 (RCQIFANI) and GP33 (KAVYNFATC) peptides were purchased from Neosystem (http://www.neomps.com/).

### Statistical analysis.

All statistical analyses were performed with Prism 4.0 (GraphPad Software, http://www.graphpad.com/). Data were analyzed with the paired Student's *t*-test assuming that the values followed a Gaussian distribution. A *p*-value of < 0.05 was considered significant.

## Supporting Information

### Accession Numbers

The GenBank (http://www.ncbi.nlm.nih.gov/Genbank/) accession numbers for the viruses and sequences discussed in this paper are HCoV-229E (AF304460), MHV-A59 (AY700211), and SARS-CoV Frankfurt-1 (AY291315).

## Abbreviations

ALT: alanine 2-oxoglutarate-aminotransferase
cDC: conventional dendritic cell
CTL: cytotoxic T lymphocyte
DC: dendritic cell
IFN: interferon
ISRE: interferon-stimulated response element
LCMV: lymphocytic choriomeningitis virus
MHV: mouse hepatitis virus
nsp1: non-structural protein 1
nt: nucleotide
pAPC: professional antigen-presenting cell
pDC: plasmacytoid dendritic cell
p.i.: post infection
